# Unraveling the Dynamics of Esophageal Motility, Esophagitis Severity, and Age in GERD Patients: A Cross-Sectional Exploration

**DOI:** 10.7759/cureus.53979

**Published:** 2024-02-10

**Authors:** Ygor R Fernandes

**Affiliations:** 1 General Surgery and Digestive Endoscopy, Escola Paulista de Medicina, Universidade Federal de São Paulo, Hospital São Paulo, São Paulo, BRA

**Keywords:** manometry, gastroesophageal reflux disease, aging, esophageal motility disorders, peptic esophagitis

## Abstract

Background: Gastroesophageal reflux disease (GERD) is characterized by prolonged exposure of the esophageal mucosa to gastric content, with esophageal motility playing a pivotal role in its pathophysiology. This study employs a cross-sectional design to investigate the interplay between esophageal motility, the severity of esophagitis, and age in individuals presenting with GERD symptoms.

Objective: The primary objective is to assess proximal and distal esophageal contractions in individuals with GERD symptoms, exploring potential correlations with the severity of esophageal lesions and age.

Methods: A total of 47 patients reporting heartburn and acid regurgitation underwent diagnostic investigations, including esophageal manometry, radiological examinations, and endoscopy. Patients were categorized into groups based on the presence and severity of esophagitis. Esophageal contractions were monitored using a manometric method at various distances from the UES after swallowing 5 mL of water.

Results: Patients with severe esophagitis (SE) exhibited a reduced distal esophageal contraction amplitude compared to those without esophagitis (WE) or with moderate esophagitis (ME). No significant age-related differences were observed in esophageal contractions. Analyses included contraction amplitude, duration, area under the curve (AUC), and propagation time.

Conclusion: This study provides insights into the nuanced relationship between esophageal motility, esophagitis severity, and age in GERD patients. The findings highlight the significance of distal esophageal contractions in SE cases, suggesting potential implications for disease progression. Age did not emerge as a significant factor influencing esophageal motility in this patient cohort.

## Introduction

Gastroesophageal reflux disease (GERD), a globally prevalent disorder, is characterized by the prolonged exposure of the esophageal mucosa to gastric contents [1]. The reduced mucosal resistance to reflux aggression, coupled with the aggressiveness of the gastric material, defines this chronic condition [1]. Symptoms, notably heartburn and acid regurgitation, significantly affect the quality of life for those affected [2]. The estimated prevalence of GERD is 13.3% of the population worldwide [2]. Risk factors for GERD include an age of 50 years or older, current smoking, use of nonsteroidal anti-inflammatory drugs, obesity (BMI >30), low socioeconomic status, and female gender [1-3].

Aging is a complex biological process characterized by progressive changes in various physiological systems, influencing the overall health of individuals [4]. Within this spectrum, the gastrointestinal tract undergoes changes, and esophageal disorders are increasingly recognized as clinically significant components of aging [4].

The aging process is linked to changes in esophageal structure and function, including changes in esophageal motility [4,5]. These age-related alterations in the amplitude and coordination of esophageal contractions may impact bolus transit efficiency, contributing to the spectrum of esophageal disorders [5]. From benign conditions like esophageal dysmotility to more severe manifestations such as esophagitis, understanding the interplay between aging and esophageal disorders is crucial for unraveling their clinical implications.

The complex relationship between aging, esophageal disease, and GERD raises important clinical questions regarding their prevalence and incidence in the aging population.

Epidemiologic data highlight an increasing burden of GERD with advancing age, underscoring the need to investigate mechanistic links between age-related esophageal changes and GERD [6].

This study aims to elucidate alterations in esophageal motility patterns in aging individuals presenting with GERD symptoms, with or without esophagitis. By addressing these questions, we seek to contribute valuable insights into the intricate interplay between aging, esophageal function, and GERD. This understanding holds the potential to refine therapeutic strategies and improve clinical interventions for this vulnerable demographic.

## Materials and methods

This prospective observational study aimed to investigate esophageal motility patterns in patients presenting with symptoms of GERD, with a specific focus on those with or WE.

Study design

This research followed a prospective observational design, employing esophageal manometry as the primary investigative method. The observational nature of the study allowed us to explore esophageal motility in a real-world setting without intervention, providing valuable insights into the natural course of GERD-related motility changes.

Participants

A total of 47 patients reporting complaints of heartburn and acid regurgitation were enrolled in the study. Inclusion criteria encompassed individuals with typical symptoms indicative of GERD, for a period longer than six months. Exclusion criteria involved individuals with atypical or silent GERD presentations, a history of previous upper gastrointestinal surgery, actual medication interfering with GERD, and known specific esophageal motor disorder. Proton pump inhibitors were stopped for at least two weeks. The study population exhibited a diverse range of age, gender, and severity of esophageal lesions. For analysis, patients were subdivided into two groups according to age: <50 years (n = 29) and >50 years (n = 18).

Diagnostic investigations

Participants underwent comprehensive diagnostic investigations for GERD, including radiological and endoscopic examinations of the esophagus. Radiological examinations yielded normal results, and endoscopy revealed varying degrees of esophagitis. The severity of esophagitis was classified according to the Los Angeles classification system.

Esophageal manometry

The assessment of esophageal motility utilized a manometric method involving an eight-channel polyvinyl catheter. This catheter, with an external diameter of 4.5 mm and an internal diameter of 0.8 mm in each channel, was equipped with side openings spaced 5 cm apart in the four proximal channels, forming 90° angles. Pressure transducers connected to a PC Polygraph HR device recorded manometric signals. Technical details of the procedure have been described elsewhere [7,8].

Procedure

The patients were studied for 24h, in a supine position after a 12h fasting. They had the catheter introduced through the nostril. The four channels of the catheter, positioned 2 cm below the upper esophageal sphincter (UES) and 5 cm, 12 cm, and 17 cm distal to the UES, were utilized to assess contractions. Five swallows of 5 mL of water were performed with a minimum 20-second interval between them. At the end of the 24h period, the patients returned to the laboratory for removal of the catheter and analysis of the data recorded. Technical details of the procedure have been described elsewhere [7,8].

Measurements and parameters

Using the Polygram Upper GI version 6.4 program, various parameters were measured, including contraction amplitude, duration, area under the curve (AUC), propagation time, and the percentage of contraction failures and peristaltic, simultaneous, and non-propagated contractions. Measurements at 2 cm and 17 cm from the UES represented the proximal and distal esophagus, respectively.

Statistical analysis

The analysis of variance (ANOVA) test, Tukey's test for multiple comparisons, and Fisher's test were employed for statistical analysis. Results were presented as mean, standard deviation, and percentage. A p-value <0.05 was considered statistically significant.

Ethical considerations

The study received approval from the Institutional Ethics Committee, and all participants provided informed consent before participation.

## Results

Endoscopy revealed normal findings in 28 patients (including 10 men) with ages ranging from 17 to 78 years (median: 41 years). Moderate esophagitis (ME) was observed in 17 patients (including six men) with ages ranging from 19 to 73 years (median: 46 years), while severe esophagitis (SE) was identified in eight patients (including five men) aged between 36 and 81 years (median: 50 years). Patients with normal endoscopy exhibited no mucosal lesions in the esophagus. Those with ME presented lesions corresponding to Los Angeles classification grades A and B, and those with SE showed grades C and D.

At 12 cm and 17 cm from the UES, the mean contraction amplitude in patients with SE was lower than that in patients WE or with ME (P < 0.05, Table 1). No differences were observed between the groups at 2 cm and 7 cm from the UES.

**Table 1:** Amplitude and duration of esophageal contractions in patients with symptoms of GERD WE (n = 28), with ME (n = 12) and with SE (n = 7), measured at 2 cm, 7 cm, 12 cm, and 17 cm from the UES after swallowing water (mean + SD) GERD, gastroesophageal reflux disease; UES, upper esophageal sphincter; WE, without esophagitis; ME, moderate esophagitis; SE, severe esophagitis

Amplitude (mmHg)				
	2 cm	7 cm	12 cm	17 cm
WE	84.8 ± 39.2	42.0 ± 25.4	80.6 ± 34.2	94.5 ± 32.9
ME	84.8 ± 41.7	39.1 ± 15.0	78.7 ± 35.93	93.5 ± 45.3
SE	95.6 ± 45.1	36.1 ± 27.5	54.1 ± 24.0	55.6 ± 26.8
Duration (seconds)				
	2 cm	7 cm	12 cm	17 cm
WE	2.6 ± 0.8	3.0 ± 0.7	3.5 ± 0.8	4.1 ± 1.1
ME	2.7 ± 0.8	2.0 ± 0.7	3.5 ± 0.7	3.9 ± 0.9
SE	2.7 ± 0.9	2.8 ± 0.8	3.3 ± 0.9	3.4 ± 1.1

At 17 cm from the UES, the AUC of contractions was significantly reduced in patients with SE compared to those WE or with ME (P < 0.01, Table 2 and Table 3), with no differences at 2 cm and 7 cm from the UES.

**Table 2:** AUC of esophageal contractions in patients with symptoms of GERD WE (n = 28), with ME (n = 12) and with SE (n = 7), measured at 2 cm, 7 cm, 12 cm, and 17 cm from the UES, after swallowing water (mean + SD) GERD, gastroesophageal reflux disease; UES, upper esophageal sphincter; AUC, area under the curve; WE, without esophagitis; ME, moderate esophagitis; SE, severe esophagitis

AUC (mm Hg x s)				
	2 cm	7 cm	12 cm	17 cm
WE	103.6 ± 53.4	76.5 ± 62.7	158.0 ± 91.2	214.3 ± 114
ME	109.7 ± 70.8	67.5 ± 29.7	145.6 ± 75.8	192.1 ± 118.7
SE	137.9 ± 95.6	63.2 ± 52.5	106.6 ± 71.2	111.1 ± 55.1

**Table 3:** Time interval between the onset of peristaltic contractions in patients with symptoms of GERD WE (n = 28), with ME (n = 12) and with SE (n = 7), measured at 2 cm, 7 cm, 12 cm, and 17 cm from the UES, after swallowing water (mean + SD) GERD, gastroesophageal reflux disease; UES, upper esophageal sphincter; WE, without esophagitis; ME, moderate esophagitis; SE, severe esophagitis

Time interval (s)			
	2 → 7 cm	7 → 12 cm	12 → 17 cm
WE	2.9 ± 1.2	1.8 ± 0.5	1.8 ± 1.0
ME	2.5 ± 0.8	1.8 ± 0.8	2.0 ± 1.0
SE	2.8 ± 0.9	1.8 ± 0.6	1.8 ± 0.6

There were no significant differences between the groups regarding the duration of contractions and the time interval between the onset of contractions (Table 1 and Table 2). Additionally, there were no disparities among the groups concerning the percentage of contraction failures and the percentage of peristaltic, simultaneous, and non-propagated contractions (Table 4 and Table 5).

**Table 4:** Percentage of contraction failures and peristaltic esophageal contractions in patients with symptoms of GERD WE (n = 28), with ME (n = 12) and with SE (n = 7), measured at 2 cm, 7 cm, 12 cm, and 17 cm from the UES, after swallowing water (mean + SD) GERD, gastroesophageal reflux disease; UES, upper esophageal sphincter; WE, without esophagitis; ME, moderate esophagitis; SE, severe esophagitis

	Contraction failures (%)				Peristaltics (%)		
	2 cm	7 cm	12 cm	17 cm	2 -> 7 cm	7 -> 12 cm	12 -> 17 cm
WE	0.5	17.1	8.6	11.0	76.7	67.6	71.4
ME	0.9	15.3	3.8	2.6	77.9	67.2	81.7
SE	0.0	18.7	8.0	9.3	73.3	61.3	73.3

**Table 5:** Percentage of simultaneous peristaltic esophageal contractions or not propagated in patients with symptoms of GERD WE (n = 28), with ME (n = 12) and with SE (n = 7), measured at 2 cm, 7 cm, 12 cm, and 17 cm from the UES, after swallowing water (mean + SD) GERD, gastroesophageal reflux disease; UES, upper esophageal sphincter; WE, without esophagitis; ME, moderate esophagitis; SE, severe esophagitis

	Simultaneous contraction (%)			Unpropagated peristalsis (%)		
	2 cm	7 cm	12 cm	2 -> 7 cm	7 -> 12 cm	12 -> 17 cm
WE	2.9	10.5	12.4	20.4	21.9	16.2
ME	5.1	13.6	10.6	17.0	19.2	7.7
SE	6.7	17.3	17.3	20.0	21.4	9.4

**Figure 1:**
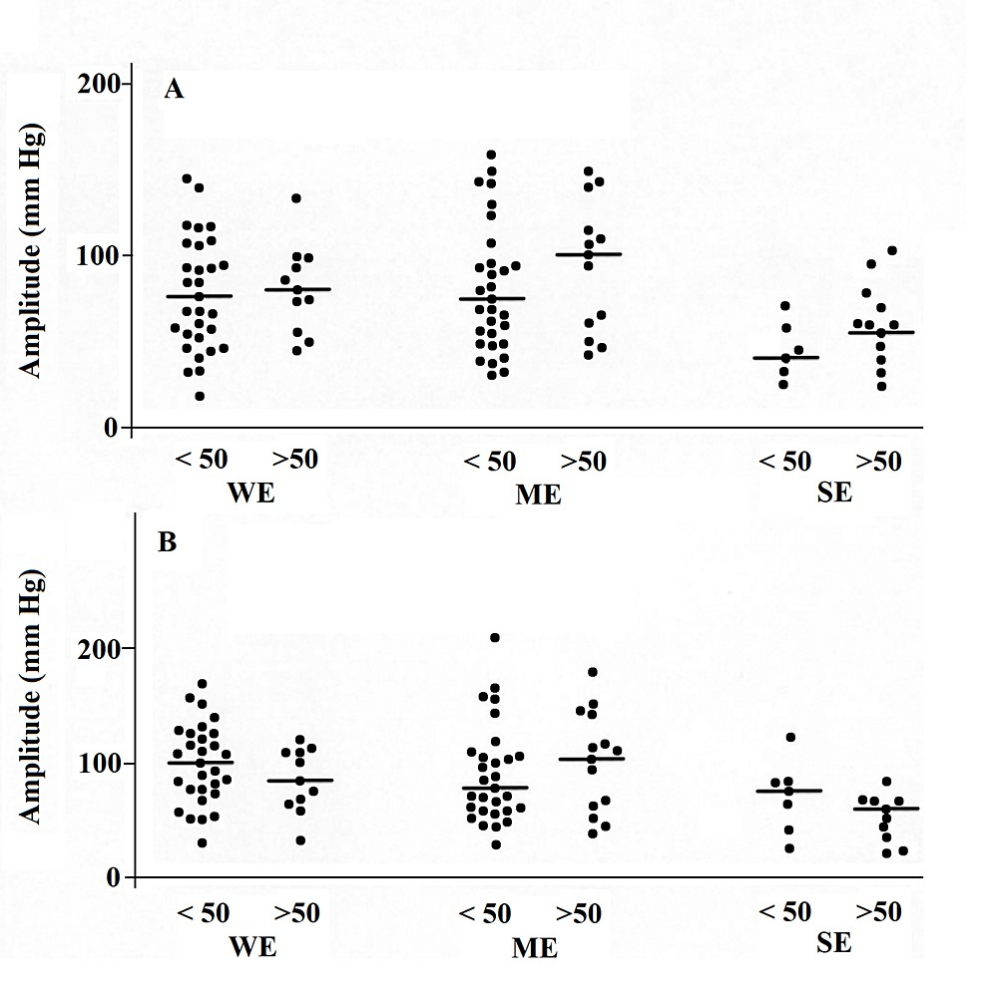
Amplitude of esophageal contractions measured at 12 cm (A) and 17 cm (B) from the UES in patients with symptoms of gastroesophageal reflux WE, with ME and with SE, aged under 50 and over 50. The horizontal lines represent the medians UES, upper esophageal sphincter; WE, without esophagitis; ME, moderate esophagitis; SE, severe esophagitis

**Figure 2:**
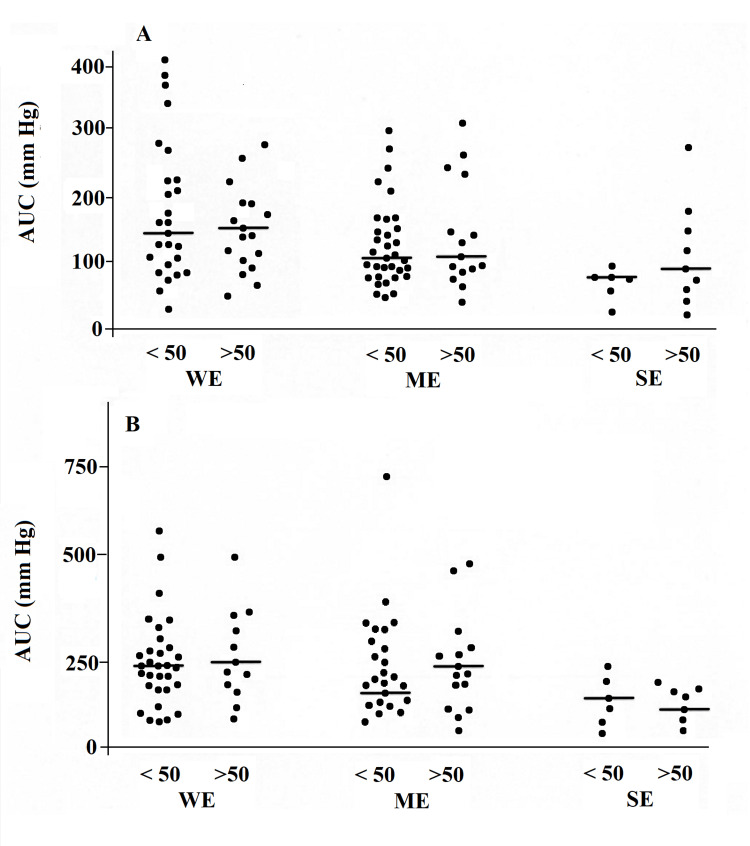
AUC of esophageal contractions measured at 12 cm (A) and 17 cm (B) from the UES in patients with symptoms of gastroesophageal reflux WE, with ME and with SE, aged below 50 years and above 50 years. The horizontal lines represent the medians UES, upper esophageal sphincter; AUC, area under the curve; WE, without esophagitis; ME, moderate esophagitis; SE, severe esophagitis

In all three groups, there were no differences between patients aged 50 and older and those under 50 years (Figure 1 and Figure 2).

## Discussion

The current study findings suggest that individuals with more severe lesions due to gastroesophageal reflux exhibit a diminished amplitude of esophageal contractions in the distal esophagus compared to those with milder esophagitis or no esophagitis. Notably, age does not appear to exert a significant influence on these contractions.

In patients with SE, the amplitude of distal esophageal contractions did not decrease to levels indicative of ineffective esophageal motility, defined as an amplitude in the distal esophagus of less than 30 mm Hg or a proportion of non-propagated contractions equal to or greater than 30% of total liquid swallowed. However, the observed amplitude of distal esophageal contractions in patients with SE was lower than that in patients without esophageal lesions or with ME, as well as lower than that in individuals studied using the same method.

Understanding the implications of decreased contraction amplitude in the distal esophagus for disease progression is crucial, particularly for patients with more severe conditions. Patients with decreased amplitude indicative of ineffective esophageal motility (<30 mm Hg) experience prolonged exposure of the esophagus to pH levels below four and difficulty in esophageal acid clearance compared to patients with normal motility [7,8]. Another study demonstrated that a significant loss of esophageal contractions leads to increased exposure of the distal esophagus to acidic volume when in the supine position, with normal clearance in the upright position [9]. Regardless of the presence of contractions in the middle and distal parts of the esophagus and irrespective of the position, the arrival of swallowed saliva in the distal esophagus is not interrupted or delayed [10]. However, when esophageal contractions are absent, saliva clearance in the supine position is prolonged [8]. It is suggested that this extended clearance time increases the risk of developing adenocarcinoma at the esophagogastric junction, where adenocarcinomas typically occur due to the interaction of saliva with acidic gastric juice, producing potentially mutagenic and carcinogenic nitrogen compounds [6,10].

Moderate ineffective esophageal motility does not affect volume or acid reflux chemical clearance [11]. Contrary to expectations, the patients with SE in this study did not exhibit a significant decrease in contractions in the distal esophagus, indicating that major impairments in clearance are not anticipated. Nevertheless, the higher intensity of esophagitis was associated with a modest decrease in distal amplitude, suggesting its potential importance in determining the severity of esophagitis. However, conflicting results question the association between ineffective esophageal motility and the presence of esophagitis [11-13]. No differences were observed between the groups in the proximal esophagus, aligning with previous findings.

No observable effect of aging on contractions was noted in this patient group. Although aging typically affects contractions, impairs transit through the esophagus, and decreases contraction amplitude, the exposure time to refluxate did not show a discernible impact in this study [4,5].

While our study primarily focused on esophageal motility and its association with the severity of esophagitis, it is crucial to acknowledge that other factors, such as dietary habits, lifestyle choices, and concurrent medications, can also influence esophageal function [12,13]. Future research should adopt a more comprehensive approach, encompassing a broader range of potential contributing factors.

In normal individuals, the transit of low or high-viscosity liquid bolus, in both the supine and seated positions, is longer in the distal part of the esophagus compared to the proximal part [14]. The amplitude of contraction is greater in the distal part of the esophagus than in the proximal part [15]. In the results of this study, patients WE or with ME had slightly higher amplitude of contractions in the distal part (17 cm from the UES) on average compared to the amplitude in the proximal part (2 cm from the UES). However, in patients with SE, the average amplitude was lower in the distal part compared to the proximal part, potentially contributing to the naturally longer transit time through the distal esophagus [16]. However, it is possible that transit in the distal esophagus does not depend on contraction amplitude, making the observation of low contraction amplitude in patients with SE, as previously noted, subject to reservations regarding cause-effect association. Other factors may be involved in both the decrease in amplitude and the severity of esophagitis. Impairment of motility in the distal esophagus and proximal stomach could determine the low amplitude of contraction, transient relaxation of the lower esophageal sphincter associated with gastroesophageal reflux, low pressure of this sphincter, and changes in the accommodation of the proximal stomach to ingested volume [16,17]. These changes, extensively studied today, are not yet completely understood in terms of their pathophysiology.

It is plausible that the decrease in amplitude of contraction in the distal esophagus is a consequence of esophagitis. This hypothesis gains support from studies indicating that amplitude and the proportion of peristaltic contractions increase after clinical treatment of reflux esophagitis, although conflicting results also exist [17-19]. The prevailing idea is that impairment of esophageal motility is irreversible, placing these patients at a higher risk for complications of gastroesophageal reflux, such as Barrett's esophagus and strictures, necessitating more effective acid suppression [18,20,21].

## Conclusions

In conclusion, our study delves into the intricate relationship between aging, esophageal motility, and GERD. The findings highlight a significant association between the severity of esophagitis due to GERD and a diminished amplitude of esophageal contractions in the distal esophagus. Importantly, age did not emerge as a significant factor influencing these contractions in our study population. The implications of reduced contraction amplitude in the distal esophagus for disease progression warrant further exploration, especially concerning the extended exposure of the esophagus to acidic pH levels and impaired clearance observed in patients with ineffective esophageal motility.

The study limitation includes a relatively small sample size of 47 patients, potentially restricting the generalizability of our results. The variability in esophageal motility among individuals with GERD and esophagitis could be influenced by diverse factors, such as genetic predisposition, lifestyle, and comorbidities. A larger and more diverse sample would enhance the robustness of our conclusions. Additionally, the study population mainly comprised patients with GERD symptoms, and the exclusion of certain patient groups, such as those with atypical or silent GERD, may limit the broader applicability of our findings. Future studies should aim to incorporate a more diverse range of GERD presentations for a comprehensive understanding.
